# Additive Renoprotection by Pioglitazone and Fenofibrate against Inflammatory, Oxidative and Apoptotic Manifestations of Cisplatin Nephrotoxicity: Modulation by PPARs

**DOI:** 10.1371/journal.pone.0142303

**Published:** 2015-11-04

**Authors:** Mai M. Helmy, Maged W. Helmy, Mahmoud M. El-Mas

**Affiliations:** 1 Pharmacology and Toxicology, Faculty of Pharmacy, Alexandria University, Alexandria, Egypt; 2 Pharmacology and Toxicology, Faculty of Pharmacy, Damanhour University, Damanhour, Egypt; The University of Manchester, UNITED KINGDOM

## Abstract

Nephrotoxicity is a major side effect for the antineoplastic drug cisplatin. Here, we employed pharmacological, biochemical, and molecular studies to investigate the role of peroxisome proliferator-activated receptors (PPARs) in cisplatin nephrotoxicity. Rats were treated with a single i.p. dose of cisplatin (5 mg/kg) alone or combined with pioglitazone (PPARγ agonist), fenofibrate (PPARα agonist), pioglitazone plus fenofibrate, or thalidomide (Tumor necrosis factor-α inhibitor; TNF-α). Cisplatin nephrotoxicity was evidenced by rises in renal indices of functional (blood urea nitrogen, BUN, and creatinine), inflammatory (TNF-α, interleukin 6, IL-6), oxidative (increased malondialdehyde, MDA, and decreased superoxide dismutase, SOD and nitric oxide metabolites, NOx), apoptotic (caspase 3), and histological (glomerular atrophy, acute tubular necrosis and vacuolation) profiles. Cisplatin effects were partly abolished upon concurrent exposure to pioglitazone, fenofibrate, or thalidomide; more renoprotection was observed in rats treated with pioglitazaone plus fenofibrate. Immunostaining showed that renal expressions of PPARα and PPARγ were reduced by cisplatin and restored to vehicle-treated values after simultaneous treatment with pioglitazone or fenofibrate. Fenofibrate or pioglitazone renoprotection remained unaltered after concurrent blockade of PPARα (GW6471) and PPARγ (GW9662), respectively. To complement the rat studies, we also report that in human embryonic kidney cells (HEK293 cells), increases caused by cisplatin in inflammatory, apoptotic, and oxidative biomarkers were (i) partly improved after exposure to pioglitazone, fenofibrate, or thalidomide, and (ii) completely disappeared in cells treated with a combination of all three drugs. These data establish that the combined use of pioglitazone and fenofibrate additively improved manifestations of cisplatin nephrotoxicity through perhaps GW6471/GW9662-insensitive mechanisms.

## Introduction

The clinical use of cisplatin, a platinum-containing antineoplastic drug, is often associated with severe side effects such as nephrotoxicity [1]. The nephrotoxic effect of cisplatin has been attributed to renal vasoconstriction, and deteriorated inflammatory, oxidative, hypoxic, and apoptotic renal profiles [2–4]. Tumor necrosis factor-α (TNF-α) is a major contributor to cisplatin nephrotoxicity through the activation of several other chemokine and cytokine modalities [5]. This view receives support from the observation that TNF-α inhibition remarkably ameliorates renal damage caused by cisplatin [6].

Peroxisome proliferator-activated receptors (PPARs) are nuclear receptors that are involved in the transcriptional modulation of diverse cellular functions such as lipid metabolism, glucose homeostasis, differentiation, and inflammation [7]. Three isoforms of PPARs (PPARα, PPARβ, PPARγ) have been characterized in body organs including the kidney [8, 9]. Recent studies highlighted a favorable effect for PPARγ agonists in diabetic and non-diabetic nephropathies [10, 11]. The PPARγ agonist pioglitazone guards against cisplatin nephrotoxicity through the upregulation of PPARγ receptor expression, which subsequently diminishes renal NF-κB, TNF-α, macrophage infiltration, and oxidative insult [12, 13]. Other studies reported a renoprotective effect for fenofibrate, PPARα agonist, against diabetic nephropathy, probably via improving oxidative stress, inflammation, and lipid profiles [14, 15].

The main objective of the current study was to investigate whether additive or synergistic renoprotection against cisplatin nephrotoxicity could develop in rats upon concurrent exposure to pioglitazone plus fenofibrate. While no information is available regarding the interaction of fenofibrate with cisplatin nephrotoxicity, pioglitazone has been shown to cause only partial improvement in nephrotoxic manifestations of cisplatin [12]. Other unique aspects of the study included (i) the use of doses of pioglitazone (2.5 mg/kg) and fenofibrate (100 mg/kg) smaller than those employed in previous studies [13, 16, 17], which might help in minimizing their potential adverse effects, (ii) determining whether the renoprotective effect of the pioglitazone/fenofibrate regimen could be replicated after the inhibition of TNF-α by thalidomide, (iii) undertaking protein expression and pharmacologic antagonist studies to assess the role of PPARs in the evoked renoprotection, and (iv) the utilization of cell culture studies (HEK293 cells) to confirm the importance of TNF-α inhibition in the renoprotective effect of the combined pioglitazone/fenofibrate regimen. To achieve these objectives, immunohistochemical analyses of renal PPARα and PPARγ expressions as well as indices of renal function, morphology, inflammation, apoptosis, and oxidative stress were evaluated.

## Materials and Methods

Male Wistar rats (180–200 g, Faculty of Pharmacy, Alexandria University, Alexandria, Egypt) were used. Experiments were performed in strict accordance with and approved by the institutional animal care and use guidelines of the Faculty of Pharmacy, Alexandria University. The manuscript does not contain clinical studies or patient data.

### Drugs

Cisplatin (OncotecPharma production, Germany), pioglitazone hydrochloride, fenofibrate, thalidomide, GW9662, GW6471 (Tocris Bioscience, Bristol, UK) and thiopental sodium (Biochemie GmbH, Vienna, Austria) were purchased from commercial vendors.

### Experimental protocols

#### PPARs/TNF-α modulation of cisplatin-induced nephrotoxicity

A total of 9 groups of rats (n = 8 each) were employed and randomly assigned to receive one of the following regimens: (i) control (saline, 1 ml /kg/day), (ii) DMSO (1 ml /kg/day), (iii) cisplatin (a single i.p. dose of 5 mg/kg) [18], (iv) cisplatin + pioglitazone (PPARγ agonist, 2.5 mg/kg/day, orally) [19], (v) cisplatin + fenofibrate (PPARα agonist, 100 mg/kg/day, orally) [20], (vi) cisplatin + pioglitazone + fenofibrate, (vii) cisplatin + pioglitazone + GW9662 (PPARγ antagonist, 1 mg/kg/day i.p) [21], (viii) cisplatin + fenofibrate + GW6471 (PPARα antagonist, 1 mg/kg/day, i.p) [22], (ix) cisplatin + thalidomide (12.5 mg/kg/day, i.p) [23]. Thalidomide was dissolved in DMSO while all other drugs were dissolved in saline. PPAR agonists and antagonists were administered daily for 5 consecutive days, starting 2 days before cisplatin injection and 3 days thereafter. At the end of experiment (72 hr after cisplatin administration), overnight fasted rats were anesthetized with thiopental sodium (50 mg/kg, i.p.). Blood samples were collected from the orbital plexus and spun at 1200 g for 10 min. The serum was aspirated and stored at -70°C till used for biochemical analyses.

Rats were euthanized with thiopental overdose, abdomen was opened, right kidney was removed, weighed, and homogenized in ice cold phosphate-buffered saline (pH = 7.4) to give 40% homogenate. The homogenate was divided into aliquots and stored at -70°C till used for the measurement of inflammatory (TNF-α and IL-6), apoptotic (caspase-3) and oxidative (MDA, SOD and NOx) parameters. For histopathological or immunohistochemical studies, left kidney was fixed in 10% formaldehyde for 18 hours at 4°C and then embedded in paraffin blocks.

#### PPARs/TNF-α modulation of cisplatin toxicity in HEK293 cells

HEK293 cells (Vaccera, Egypt) were cultured in Dulbecco’s modified Eagle’s medium (DMEM) with Glutamax-1, sodium pyruvate, and 1 mg/ml glucose supplemented with 50 μg/ml streptomycin, 50 U/ml penicillin, 2 mm glutamine, and 10% (v/v) fetal calf serum. Cells (1x10^6^) were seeded in T-75 cm^2^ flask and incubated at 37°C in 90% air/10% CO_2_ for 3 days to reach 70% confluence [24]. Cells were treated with one of the following treatments for 24 hr (5 replicates): (i) DMSO, (ii) cisplatin 14 μM [24], (iii) cisplatin + pioglitazone 10 μM [25], (vi) cisplatin + fenofibrate 20 μM [26], (v) cisplatin + fenofibrate + pioglitazone, (vi) cisplatin + thalidomide 100 μM [27], (vii) cisplatin + fenofibrate + pioglitazone + thalidomide. Cells were washed with phosphate buffered saline (PBS) and trypsinized. Cell pellets were re-suspended in 1 ml PBS and cell lysates were obtained using Cell Lysis Buffer 10X (Cell Signaling Technology Cat No. 9803) as described by the manufacturer [28], divided into 4 aliquots and stored at -70°C till used for biochemical determinations (TNF-α, IL-6, caspase-3, and SOD).

### Biochemical analyses

The determination of serum urea and creatinine was carried out by the Randox^®^ assay kit (Randox Laboratories Ltd., United Kingdom). The measured urea levels were used for the calculation of blood urea nitrogen (BUN). Renal levels of MDA [29], NOx metabolites [30] and SOD activity [31, 32] were assayed spectrophotometrically. The Enzyme-Linked Immunosorbent Assay (ELISA) was used for measuring renal TNF-α (Invitrogen incorporations, CA), IL-6 (Abnova, Taiwan) and caspase-3 (WKEA MED supplies Corp., NY, USA) as instructed by the manufacturer and used in our previous studies [33,34].

### Histopathological studies

**Fig 1:**
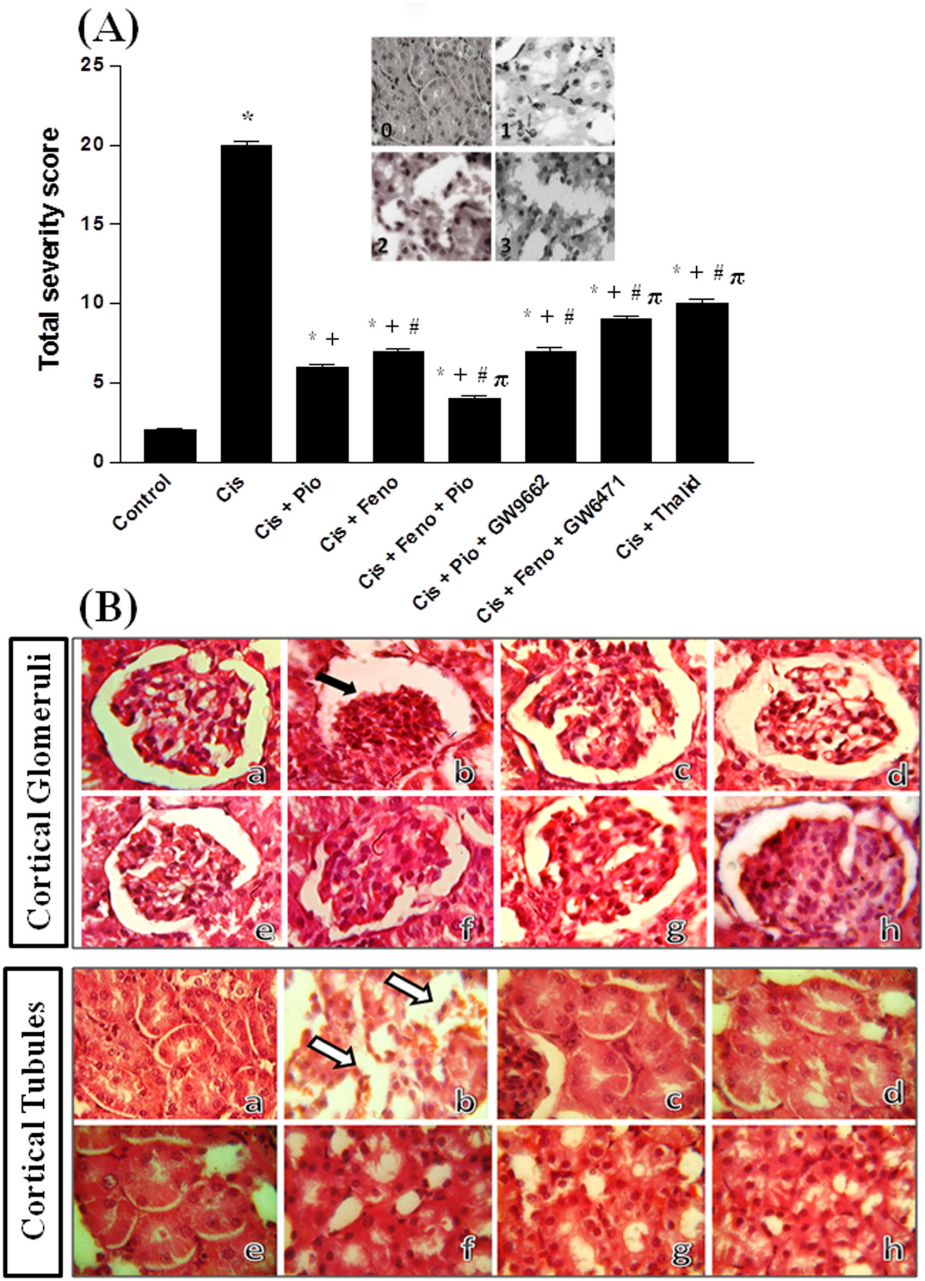
Panel A, the total severity score for tubular damage obtained from H&E stained kidney sections (×400): score 0: normal histology, score 1: tubular cell swelling, brush border loss with up to 1/3 of the tubular profile showing nuclear loss, score 2: from 1/3 to 2/3 of the tubular profile showing nuclear loss, and score 3: more than 2/3 of the tubular profile showing nuclear loss. Shown also in panel A the graphical presentation of the total severity score for tubular necrosis of different treated groups. *, ^+^, ^#^ and ^π^ denote significant difference (P<0.05) vs. control, cisplatin, cisplatin+pioglitazone and cisplatin+fenofibratevalues, respectively. **Panel B**, hematoxylin and eosin stained photomicrographs (×100 and ×400) of kidneys obtained from rats treated with saline (control, image a), cisplatin (5mg/kg, i.p., image b), cisplatin+pioglitazone (2.5 mg/kg/day, orally, image c), cisplatin+fenofibrate (100 mg/kg/day, orally, image d), cisplatin+pioglitazone+fenofibrate (e), cisplatin+pioglitazone+GW9662 (1 mg/kg/day, i.p, image f), cisplatin+fenofibrate+GW6471 (1 mg/kg/day, i.p, image g) and cisplatin+thalidomide (12.5 mg/kg/day, i.p, image h). The black arrow shows glomerular atrophy whereas white arrows point to acute tubular necrosis, marked dilation of proximal convoluted tubules with vacuolation.

The haematoxylin and eosin (H & E) stain was used for the detection of histopathological changes in renal tissues. Tubular necrosis was quantified as described in previous studies including ours [35, 36]. Ten random kidney sections (x100 &×400) were examined and a score from 0 to 3 was given according to the following arbitrary scale (Fig 1A, inset): (i) score 0: normal histology, (ii) score 1: tubular cell swelling, brush border loss with up to 1/3 of the tubular profile showing nuclear loss, (iii) score 2: from 1/3 to 2/3 of the tubular profile showing nuclear loss, and (iv) score 3: more than 2/3 of the tubular profile showing nuclear loss. All 10 scores were added to give the total necrosis score for each kidney.

### Immunostaining

The technique was performed as described previously [37–39]. Kidney sections (5 μm) were deparaffinized in xylene, rehydrated in ethanol (100%, 95%, and 70%), rinsed with phosphate-buffered saline (PBS), and drained. Antigenic determinants in cells were unblocked by 20-min incubation in citrate buffer (95–98°C, pH 6, Thermo Scientific^®^ Germany) and rinsed with 1X TBST (50 mM Tris/HCl, pH 7.4, 150 mM NaCl, 0.1% Tween 20, Thermo Scientific^®^ Germany). Endogenous peroxidases were blocked by adding 3% hydrogen peroxide. A universal protein block was applied for 20 minutes. The appropriate primary monoclonal antibodies for PPARα and PPARγ (Bioss^®^ USA) were diluted as instructed by the manufacturer and applied to the slides for 45 minutes at 37°C. Slides were washed with 1X TBST, rinsed, and incubated for 30 min with secondary antibody (polyvalent HRP detection kit, Spring Bioscience^®^ Pleasanton, CA). The chromogen 3, 3’-Diaminobenzidine (DAB) was prepared and applied as instructed by the manufacturer for protein visualization. Slides were counterstained with hematoxylin and dipped in ascending concentrations of alcohol and then xylene. Immunohistochemical signals of PPARα and PPARγ were quantified by the Image J software (version 1.45s) together with computer-assisted microscopy and grayscale thresholding as previously described [37–39].

### Statistical analysis

Data are expressed as means ± S.E.M. Normal distribution was checked using column statistics (modified Kolmogorov–Smirnov test). Because data were normally distributed and included one independent variable (drug treatments) and multiple comparisons (more than two experimental groups), statistical significance was tested with the one-way analysis of variance (ANOVA) followed by the Bonferroni post-hoc test. Analysis were performed using GraphPad Prism, software release 3.02. Probability levels less than 0.05 were considered significant.

## Results

### PPARs/TNF-α modulation of cisplatin-induced renal structural damage

Histopathological changes caused by cisplatin alone or in combination with various renoprotective protocols are presented in Fig 1 (panel B). Compared to control group (Fig 1a), kidneys of cisplatin-treated rats showed glomerular atrophy, acute tubular necrosis and vacuolation (Fig 1b). In kidneys obtained from rats treated with cisplatin along with pioglitazone (Fig 1c), fenofibrate (Fig 1d), or thalidoamide (Fig 1h), minimal glomerular or tubular atrophy or necrotic changes were demonstrated. The blockade of PPARγ (GW9662, Fig 1f) or PPARα (GW6471, Fig 1g) failed to modify the beneficial morphological effects of pioglitazone and fenofibrate, respectively. Near-normal glomerular and tubular structures were observed when the pioglitazone/fenofibrate regimen was administered to cisplatin-treated rats (Fig 1e). Similarly, the tubular necrosis score was significantly reduced when the pioglitazone/fenofibrate regimen was administered to cisplatin-treated rats (Fig 1, panel A).

### PPARs/TNF-α modulation of cisplatin-induced renal biochemical changes

**Fig 2:**
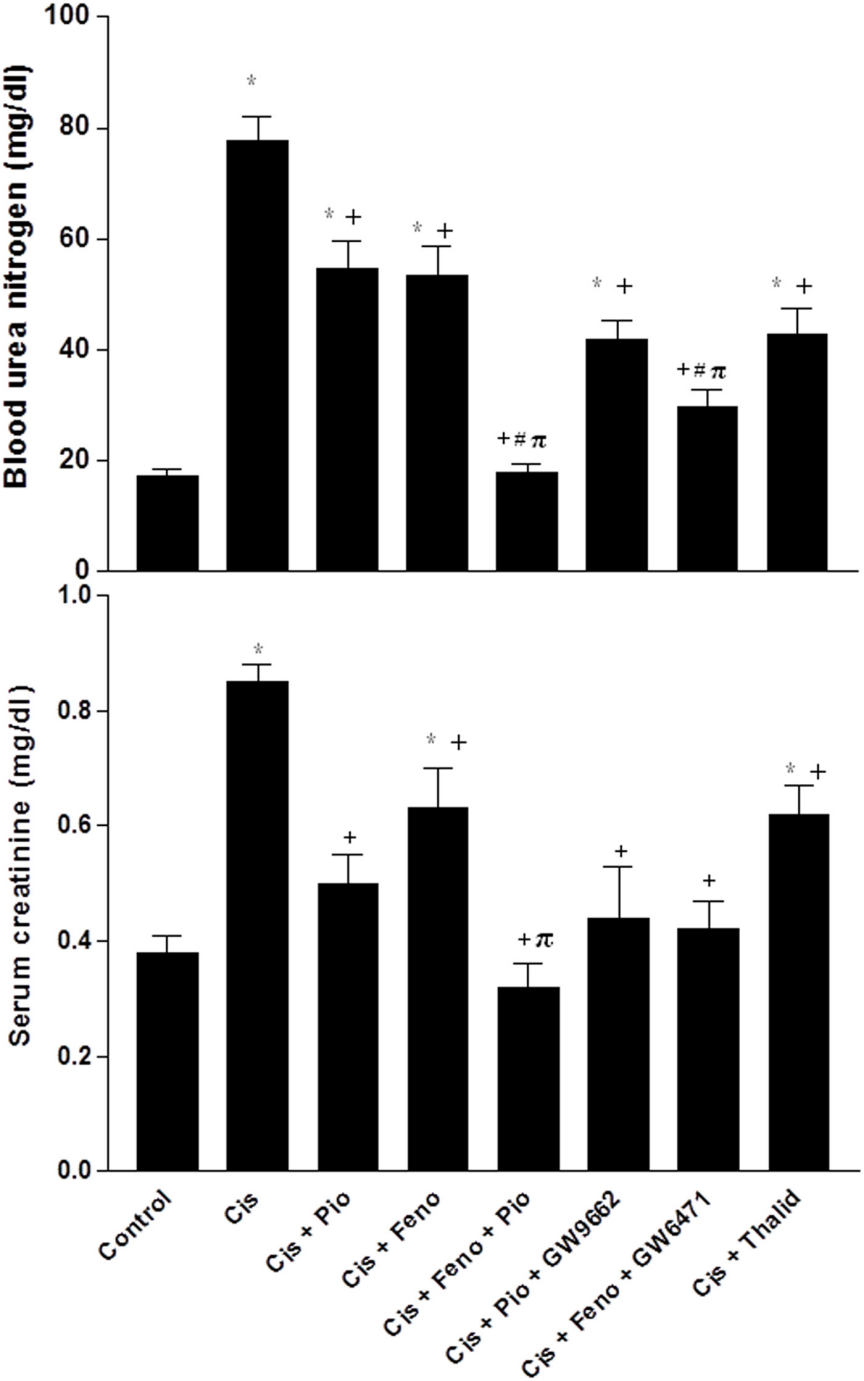
Blood urea nitrogen and serum creatinine concentration obtained from rats treated with saline (control), cisplatin (Cis, 5mg/kg, i.p.), cisplatin+pioglitazone (PIO, 2.5 mg/kg/day, orally), cisplatin+fenofibrate (Feno, 100 mg/kg/day, orally), cisplatin+pioglitazone+fenofibrate, cisplatin+pioglitazone+GW9662 (1 mg/kg/day, i.p), cisplatin+fenofibrate+GW6471 (1 mg/kg/day, i.p) and cisplatin+thalidomide (Thalid, 12.5 mg/kg/day, i.p). All drugs were administered 2 days before cisplatin and continued for 3 days thereafter. Values are means±S.E.M. of 8 observations. *, ^+^, ^#^ and ^π^ denote significant difference (P<0.05) vs. control, cisplatin, cisplatin+pioglitazone andcisplatin+fenofibratevalues, respectively.

**Fig 3:**
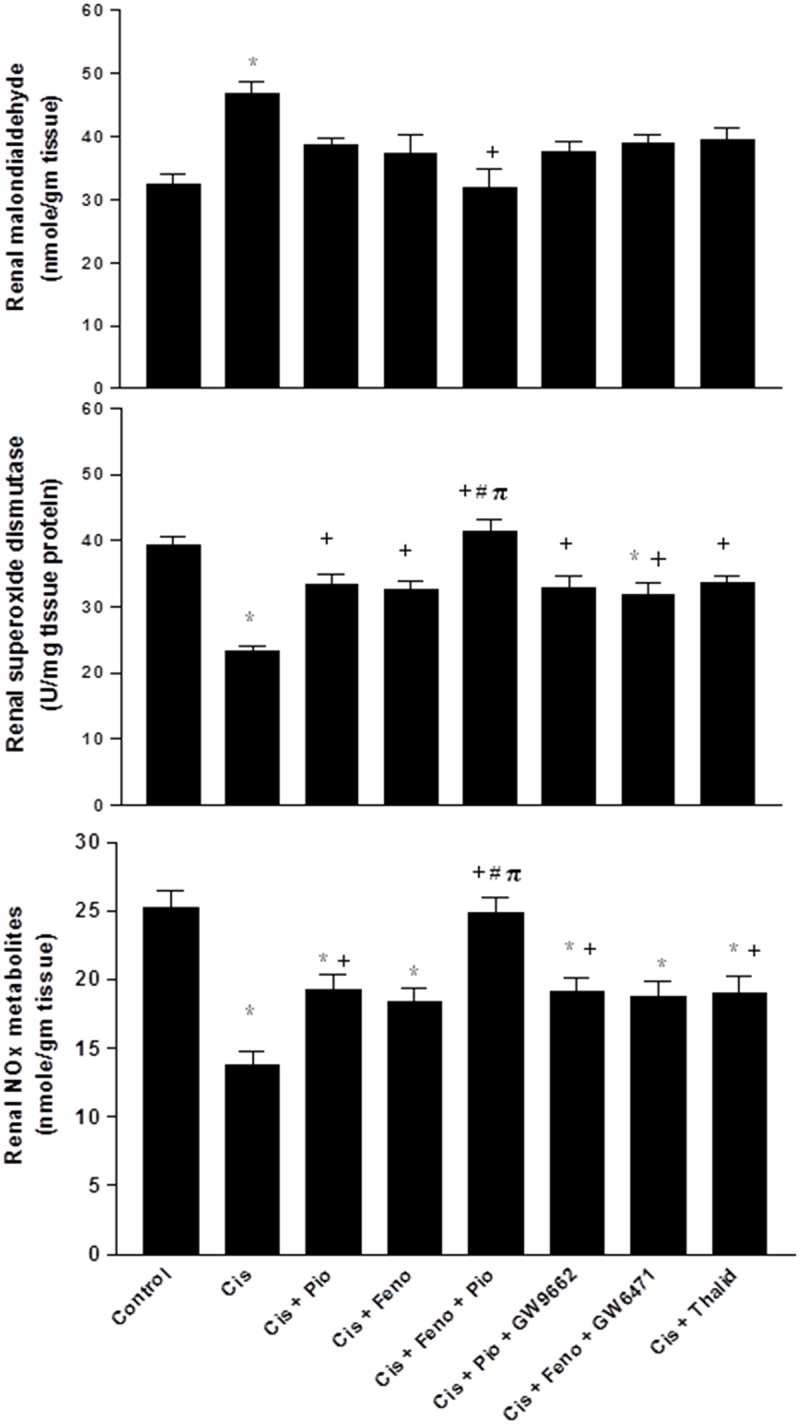
Renal malondialdehyde, superoxide dismutase, and NOx metabolites in kidney homogenates obtained from rats treated with saline (control), cisplatin (Cis, 5mg/kg, i.p.), cisplatin+pioglitazone (PIO, 2.5 mg/kg/day, orally), cisplatin+fenofibrate (Feno, 100 mg/kg/day, orally), cisplatin+pioglitazone+fenofibrate, cisplatin+pioglitazone+GW9662 (1 mg/kg/day, i.p), cisplatin+fenofibrate+GW6471 (1 mg/kg/day, i.p) and cisplatin+thalidomide (Thalid, 12.5 mg/kg/day, i.p). All drugs were administered 2 days before cisplatin injection and continued for 3 days thereafter. Values are means±S.E.M. of 8 observations. *, ^+^, ^#^ and ^π^ denote significant difference (P<0.05) vs. control, cisplatin, cisplatin+pioglitazone andcisplatin+fenofibratevalues, respectively.

**Fig 4:**
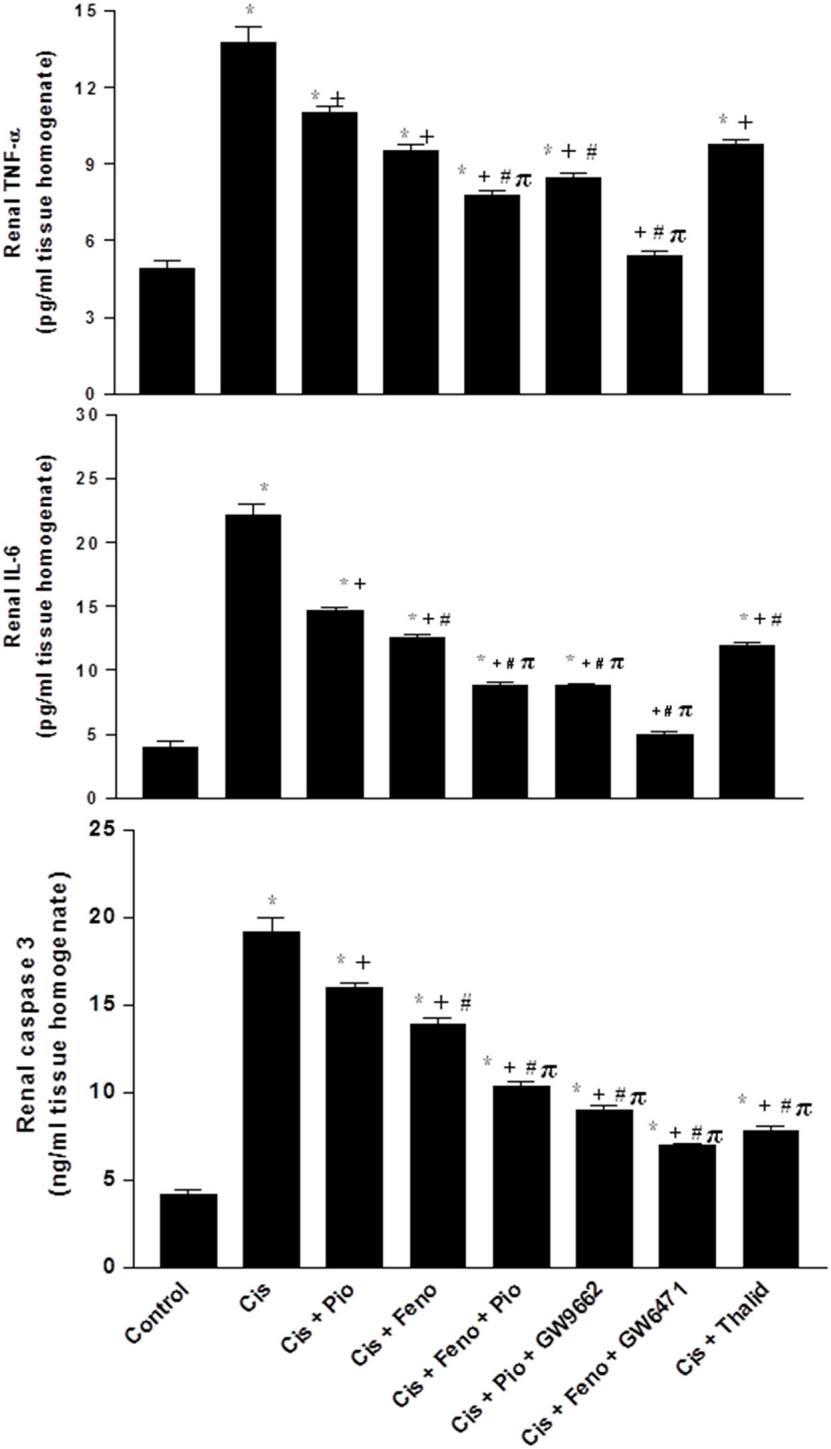
Renal TNF-α, IL-6, and caspase-3 in kidney homogenates obtained from rats treated with saline (control), cisplatin (Cis, 5mg/kg, i.p.), cisplatin+pioglitazone (PIO, 2.5 mg/kg/day, orally), cisplatin+fenofibrate (FEno, 100 mg/kg/day, orally), cisplatin+pioglitazone+fenofibrate, cisplatin+pioglitazone+GW9662 (1 mg/kg/day, i.p), cisplatin+fenofibrate+GW6471 (1 mg/kg/day, i.p) and cisplatin+thalidomide (Thalid, 12.5 mg/kg/day, i.p). All drugs were administered 2 days before cisplatin injection and continued for 3 days thereafter. Values are means±S.E.M. of 8 observations. *, ^+^, ^#^ and ^π^ denote significant difference (P<0.05) vs. control, cisplatin, cisplatin+pioglitazone andcisplatin+fenofibratevalues, respectively.

The effects of cisplatin on renal functional, oxidative, inflammatory, and apoptotic markers in the absence and presence of pharmacologic modulators of PPARs and TNF-α are shown in Figs 2–4. The levels of BUN (17.1±1.51 vs. 17.12±1.57 mg/dl) and serum creatinine (0.38±0.03 vs. 0.37±0.03 mg/dl) in rats treated with saline or DMSO were not statistically different. Acute cisplatin administration (5mg/kg, i.p) caused significant increases in serum BUN and creatinine compared with saline-treated animals (Fig 2). These effects of cisplatin were partly reduced when rats were treated concurrently with pioglitazone (PPARγ agonist), fenofibrate (PPARα agonist), or thalidomide (TNF-α inhibitor). Moreover, the rises in serum urea and creatinine seen in cisplatin-treated rats were virtually abolished when rats were concurrently treated with the combined pioglitazone/fenofibrate regimen (Fig 2).

Cisplatin also caused significant increases in renal contents of MDA, TNF-α, IL-6, and caspase-3, and decreases in renal NOx and SOD activity (Figs 3 and 4). Such oxidative, inflammatory, and apoptotic effects of cisplatin were reduced in rats after concomitant activation of PPARγ (pioglitazone) or PPARα (fenofibrate) or TNF-α inhibition (thalidomide). Moreover, the cisplatin-evoked changes in the renal oxidative profile disappeared in rats treated concurrently with the pioglitazone/fenofibrate regimen (Fig 3). The latter combination also reduced inflammatory (TNF-α and IL-6) and apoptotic (caspase-3) markers to levels that were significantly lower than the those caused by individual treatments but still remained higher than corresponding values in control rats (Fig 4). The beneficial renal effects of pioglitazone or fenofibrate were still manifest after the concurrent blockade of their respective receptors with GW9662 and GW6471 (Figs 2–4).

### PPARs/TNF-α modulation of the cisplatin-induced changes in the renal protein expression of PPARs

**Fig 5:**
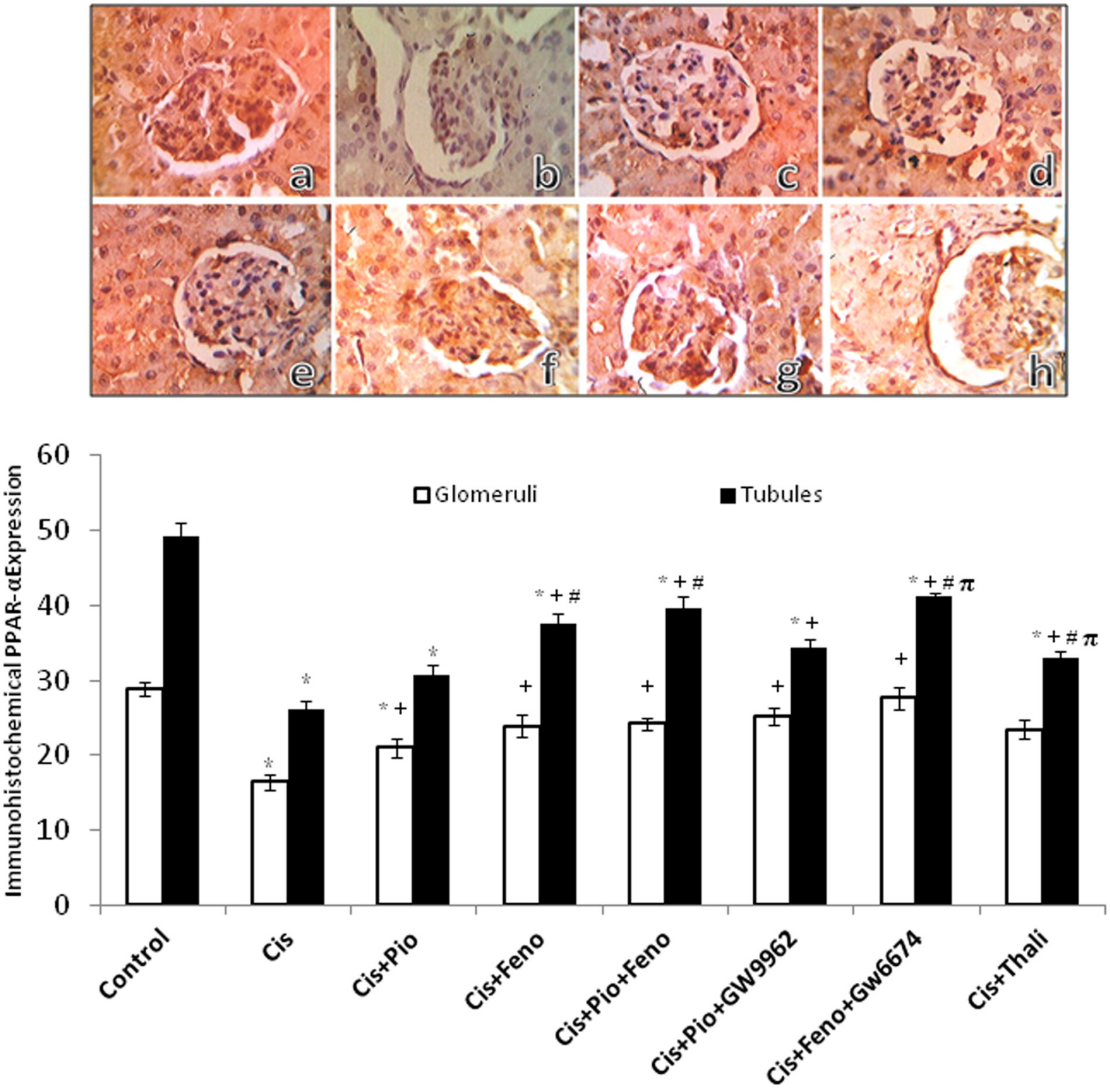
Immunohistochemical PPAR-α receptor expression in renal cortical glomeruli and medullary tubules obtained from rats treated with saline (control), cisplatin (Cis, 5mg/kg, i.p.), cisplatin+pioglitazone (PIO, 2.5 mg/kg/day, orally), cisplatin+fenofibrate (Feno, 100 mg/kg/day, orally), cisplatin+pioglitazone+fenofibrate, cisplatin+pioglitazone+GW9662 (1 mg/kg/day, i.p), cisplatin+fenofibrate+GW6471 (1 mg/kg/day, i.p) and cisplatin+thalidomide (Thalid, 12.5 mg/kg/day, i.p). All drugs were administered 2 days before cisplatin injection and continued for 3 days thereafter. Values are means±S.E.M. of 8 observations.*, ^+^, ^#^ and ^π^ denote significant difference (P<0.05) vs. control, cisplatin, cisplatin+pioglitazone and cisplatin+fenofibratevalues, respectively. Representative images of immunostained tissues are also shown (×400, A, control; B, cisplatin; C, cisplatin + pioglitazone; D, cisplatin + fenofibrate; E, cisplatin + pioglitazone + fenofibrate; F, cisplatin + pioglitazone + GW9662; G, cisplatin + fenofibrate + GW6471; H, cisplatin + thalidomide).

**Fig 6:**
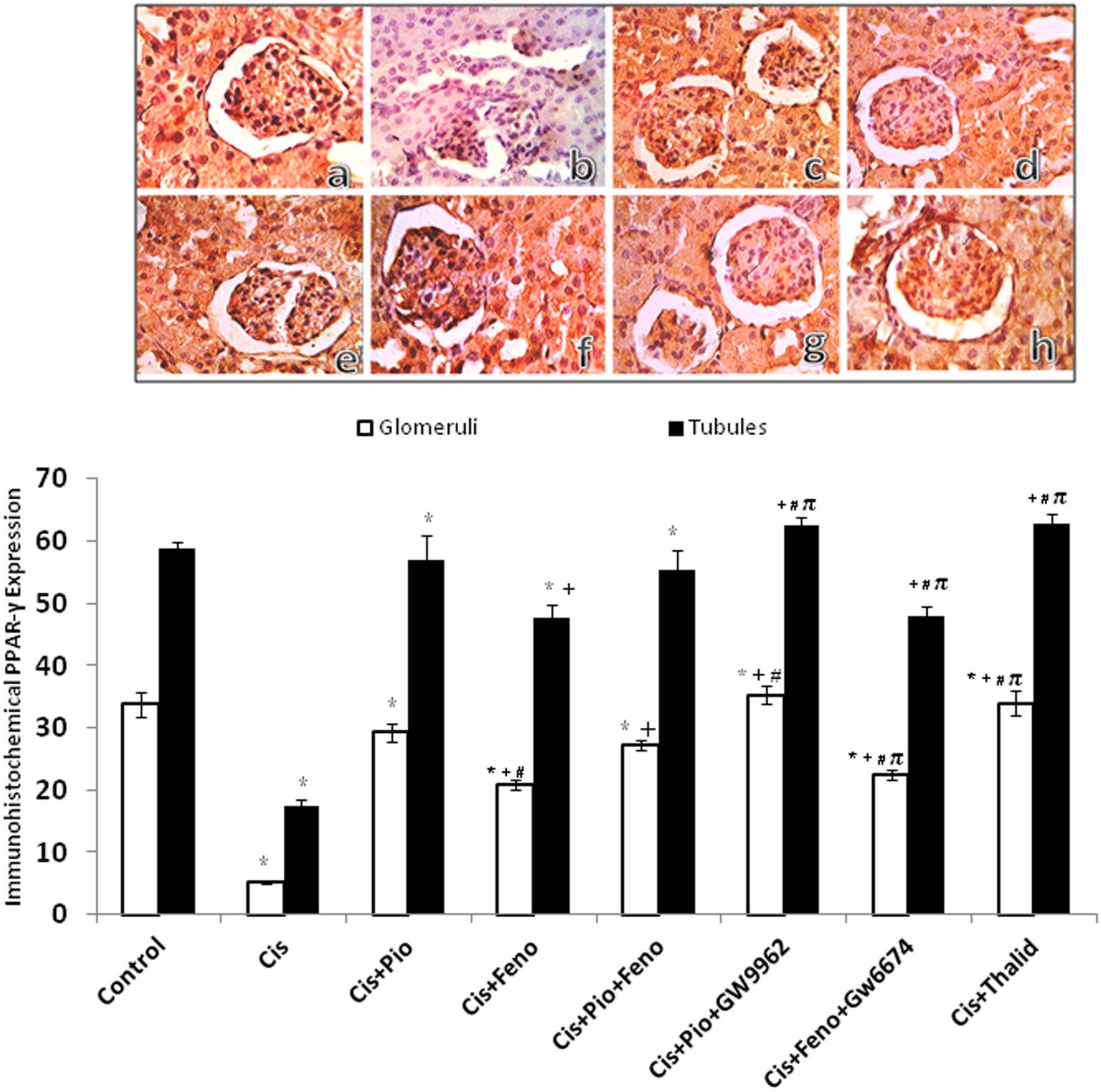
Immunohistochemical PPAR-γ receptor expression in renal cortical glomeruli and medullary tubules obtained from rats treated with saline (control), cisplatin (Cis, 5mg/kg, i.p.), cisplatin+pioglitazone (PIO, 2.5 mg/kg/day, orally), cisplatin+fenofibrate (Feno, 100 mg/kg/day, orally), cisplatin+pioglitazone+fenofibrate, cisplatin+pioglitazone+GW9662 (1 mg/kg/day, i.p), cisplatin+fenofibrate+GW6471 (1 mg/kg/day, i.p) and cisplatin+thalidomide (Thalid, 12.5 mg/kg/day, i.p). All drugs were administered 2 days before cisplatin injection and continued for 3 days thereafter. Values are means±S.E.M. of 8 observations.*, ^+^, ^#^ and ^π^ denote significant difference (P<0.05) vs. control, cisplatin, cisplatin+pioglitazone and cisplatin+fenofibratevalues, respectively. Representative images of immunostained tissues are also shown (×400, A, control; B, cisplatin; C, cisplatin + pioglitazone; D, cisplatin + fenofibrate; E, cisplatin + pioglitazone + fenofibrate; F, cisplatin + pioglitazone + GW9662; G, cisplatin + fenofibrate + GW6471; H, cisplatin + thalidomide).

Figs 5 and 6 depict changes evoked by different drug regimens in the immunohistochemical protein expression of PPARα and PPARγ in glomerular and tubular sites. Compared to the control group, treatment with cisplatin caused significant decreases in the renal expression of PPARα (Fig 5) and PPARγ (Fig 6). The cisplatin effects were partly and similarly reversed in rats treated concurrently with pioglitazone, fenofibrate, or their combination. Likewise, TNF-α inhibition by thalidoamide significantly increased PPAR expression when given along with cisplatin. Representative images of the immunohistochemical signals of PPARα and PPARγ receptors in kidneys are shown in Figs 5 and 6, respectively.

#### PPARs/TNF-α modulation of cisplatin toxicity in HEK293 cells

**Fig 7:**
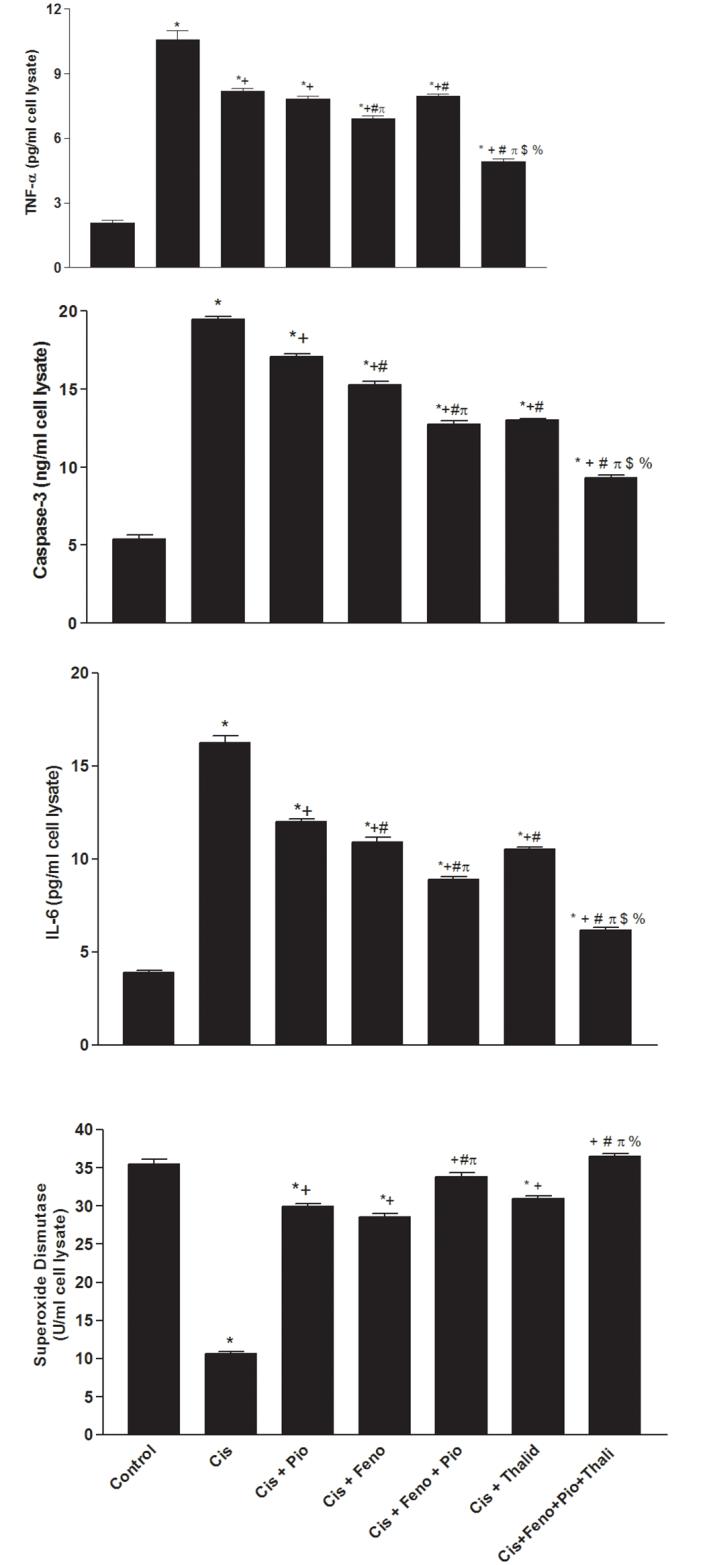
Levels of TNF-α, caspase-3, IL-6, and superoxide dismutase in HEK293 cells lysates treated with vehicle, cisplatin (Cis, 10 μM), cisplatin+pioglitazone (PIO, 10 μM), cisplatin+fenofibrate (Feno, 10 μM), cisplatin+pioglitazone+fenofibrate, cisplatin+thalidomide (Thalid, 10 μM), and cisplatin+pioglitazone+fenofibrate+thalidomide. All drugs were incubated for 24 hr. Values are means±S.E.M. of 5 replicas. *, ^+^, ^#^, ^π^, ^$^ and ^%^ denote significant difference (P<0.05) vs. control, cisplatin, cisplatin+pioglitazone, cisplatin+fenofibrate cisplatin+pioglitazone+fenofibrate and cisplatin+thalidomide values, respectively.

Compared with control (DMSO-treated) values, the incubation of HEK cells with cisplatin (14 μM) caused significant decreases in cellular SOD activity and increases in TNF-α, IL-6, caspase-3 levels (Fig 7). These effects of cisplatin were less manifest in cells co-treated with pioglitazone (10 μM), fenofibrate (20 μM), or thalidomide (100 μM), but remained significantly different from respective control values. The incubation of HEK cells with a mixture of all 3 drugs (pioglitazone, fenofibrate, and thalidomide) fully abolished the detrimental cisplatin effects on biomarkers of the inflammatory, oxidative, and apoptotic profiles (Fig 7).

## Discussion

The current study is the first to report on the effect of concurrent administration of relatively small doses of pioglitazone and fenofibrate on cisplatin-induced acute renal failure in rats. The pioglitazone/fenofibrate regimen virtually blunted most of the deleterious effects of cisplatin on functional, oxidative stress, inflammatory, apoptotic, and structural renal profiles. These beneficial effects of the pioglitazone/fenofibrate regimen might relate to the reversal of the cisplatin-evoked reductions in the protein expression of PPARα and PPARγ in renal tissues. Nonetheless, the continued demonstration of the renoprotective effect of pioglitazone and fenofibrate after PPARs blockade implies the possible involvement of PPAR-independent or GW6471/GW9662-insensitive pathways in the evoked renoprotection.

Consistent with previous reports [12, 13], we found that pioglitazone improved biochemical (serum BUN and creatinine) and structural (glomerular atrophy and tubular necrosis) renal derangements caused by acute cisplatin. Whereas the present study is the first to report on the renoprotective effect of fenofibrate against cisplatin nephrotoxicity, a similar advantageous effect for fenofibrate has been demonstrated in other animal models such as diabetic nephropathy [15]. More importantly, we report that unlike the limited improvement in renal function caused by the single use of pioglitazone or fenofibrate, the co-administration of the two drugs elicited additive protection against cisplatin nephrotoxicity and restored renal function to near-control levels. Interestingly, the doses of pioglitazone and fenofibrate employed in the current study were relatively smaller than those reported in earlier studies [13, 17]. As a consequence, the diminution of potential adverse events usually seen with these drugs is likely.

Data from previous studies established key roles for inflammatory and oxidative pathways in acute nephrotoxicity caused by cisplatin [3, 33, 34, 40]. Based on these reports, cisplatin nephrotoxicity observed in the current study can be attributed to the concomitant deterioration in the antioxidant propensity (reduced SOD) and elevations in inflammatory (TNF-α and IL-6), lipid peroxidation (MDA), and apoptotic biomarkers (caspase-3) in renal tissues. By the same token, the reversal of these latter effects upon simultaneous exposure to pioglitazone and fenofibrate may account, at least partly, for the developed renoprotection. Indeed, TNF-α has been shown to contribute to cisplatin-induced renal injury through the activation of a network of chemokines and cytokines [5]. Moreover, TNF-α activates and downregulates a number of apoptotic and antiapoptotic proteins, respectively [41, 42]. Caspase-3, an apoptosis inducer, cleaves and activates poly (ADP-ribose) polymerase which leads to DNA fragmentation [43].

In line with previous reports [3, 44], cisplatin administration resulted in significant decreases in renal NOx, a measure of the NOS-generated NO [45, 46]. Evidence suggests that the increase in free radicals, e.g. superoxide anion, would inactivate bioactive NO resulting in decreased vasodilation, reduced glomerular filteration rate and subsequent nephrotoxicity [3, 44]. L-Arginine, the NO precursor, has been shown to improve the cisplatin-induced nephrotoxicity [3], thereby confirming the important role of reduced NO bioavailability in cisplatin nephrotoxicity. This view gains support from the current observation that the renoprotective effect of the pioglitazone/fenofibrate regimen was accompanied by increased renal NOx. It is notable, however, that paradoxical increases in renal iNOS expression and NOx accumulation have been noted in cisplatin nephrotoxicity [47, 48]. More studies are apparently needed to define more clearly the role of NOS/NO signaling in cisplatin nephrotoxicity.

Molecular evidence for the expression of PPARα and PPARγ in renal tissues has been documented [8, 10, 11]. Physiologically, PPARα functions to maintain renal balance between energy production and utilization through the regulation of genes involved in β-oxidation of fatty acids [49], whereas PPARγ improves glucose tolerance and exhibits anti-proteinuric, vasculoprotective, antiinflammatory and antifibrotic effects [50]. In this study, two approaches were pursued to investigate whether PPARs contribute to pioglitazone and fenofibrate renoprotection. The first approach employed the immunohistochemical analysis to determine the protein expression of PPARs in cortical glomeruli and medullary tubules. The data showed that cisplatin nephrotoxicity was paralleled with reduced abundance of renal PPARγ and PPARα, which was partly reversed upon co-treatment with pioglitazone and/or fenofibrate. Considering the favorable renal effects of PPARs [51, 52] and worsened renal profile in animals lacking the PPAR gene [53], it is possible that the enhanced protein expression of renal PPARγ and PPARα might account for the renoprotection elicited by PPAR activation in cisplatin nephrotoxicity.

Pharmacologic receptor antagonism was the second approach we adopted to verify the involvement of PPARs in renoprotection caused by pioglitazone plus fenofibrate. Contrary to our expectations, the capacity of pioglitazone or fenofibrate to favorably affect functional, inflammatory, oxidative, and apoptotic profiles of cisplatin nephrotoxicity were preserved under conditions of PPARγ (GW9662) and PPARα (GW6471) blockade, respectively. Remarkably, the doses of GW9662 and GW6471 employed in this study have been shown adequate for blocking their respective PPARs [21, 22]. Therefore, our pharmacological data argue against the involvement of PPARs in pioglitazone or fenofibrate renoprotection. That said, the possibility that the lack of effects of GW9662 or GW6471 might be due to inadequate receptor blocking activity cannot be unequivocally excluded particularly in view of the continued elevations in renal expressions of PPARα or PPARγ in the presence of their antagonists.

It is notable, however, that other than PPAR activation, glitazones produce several pleiotropic effects that might explain their ability to improve oxidative insult, endothelium dysfunction, microalbuminuria, and lipid and platelets abnormalities [54, 55]. Further, pioglitazone decreases the proinflammatory cytokines IL-6 and 8 in endometrial stromal cells [56] and attenuates morphine withdrawal syndrome via PPAR-independent mechanisms [57]. Fenofibrate also elicits some PPAR-independent effects such as growth suppression in human endothelial cell and hepatocellular carcinoma [58, 59]. Together, in view of the current protein expression and receptor antagonist data, more studies are necessary to precisely define the role of PPARs in renoprotection offered by pioglitazone plus fenofibrate against cisplatin toxicity.

It is important to comment on the role of TNF-α, a major upstream activator of several chemokine/cytokine pathways [5], in protecting against cisplatin nephrotoxicity. This presumption receives support from several observations of the current study. First, the favorable renal effect of the pioglitazone/fenofibrate regimen was coupled with the reversal of the cisplatin-evoked increases in renal TNF-α. Second, the improved renal function and related inflammatory, apoptotic, and oxidative cascades were also demonstrated after treatment with the TNF-α inhibitor thalidomide in this and previous [60] studies. Third, results obtained from the rat studies were complemented by our cell culture studies in HEK293 cells, which showed that the detrimental inflammatory, apoptotic, and oxidative insults caused by cisplatin were (i) partially improved after single exposure to pioglitazone, fenofibrate, or thalidomide, and (ii) completely disappeared in cells treated with a combination of all three drugs. These data point out to the importance of TNF-α inhibition in the improved renal profile caused by pioglitazone plus fenofibrate and highlight the beneficial effect of combining PPAR agonists and thalidomide within this context. Another therapeutic advantage for thalidomide relates probably to its ability to enhance the antitumor and antiangiogenic activities of cisplatin [61]. Thus, the therapeutic benefits of thalidomide might not be limited to the preservation of the renal function, but also to boosting the antiproliferative effect of cisplatin.

In conclusion, the current experimental study is the first to report on the additive beneficial effects of relatively small doses of fenofibrate and pioglitazone, against cisplatin-induced acute renal failure in rats and emphasizes the key role for TNF-α inhibition in this interaction. The normalization of biochemical, morphological, inflammatory, oxidative, and apoptotic renal profiles contribute to the enhanced renoprotective effect of the pioglitazone/fenofibrate regimen. This advantageous renal effect of PPAR activators along with their capacity to enhance the growth inhibitory effect of cisplatin [12] and offset the development of cancer chemoresistance [62] might boost the anticipated benefits of using these drugs along with cisplatin in clinical practice. Notably, the advantageous renoprotective effects of the pioglitazone/fenofibrate regimen in nephropathies caused by other insults, e.g. diabetes [63], cannot be overlooked.
